# Play Behavior in Wolves: Using the ‘50:50’ Rule to Test for Egalitarian Play Styles

**DOI:** 10.1371/journal.pone.0154150

**Published:** 2016-05-11

**Authors:** Jennifer L. Essler, Simona Cafazzo, Sarah Marshall-Pescini, Zsófia Virányi, Kurt Kotrschal, Friederike Range

**Affiliations:** 1 Wolf Science Center, Ernstbrunn, Austria; 2 Comparative Cognition, Messerli Research Institute, University of Veterinary Medicine Vienna, Medical University of Vienna and University of Vienna, Vienna, Austria; 3 Department of Behavioral Biology, University of Vienna, Vienna, Austria; University of Lethbridge, CANADA

## Abstract

Social play is known as a cooperative interaction between individuals involving multiple mechanisms. However, the extent to which the equality of individuals’ play styles affects the interaction has not been studied in many species. Dyadic play between wolf puppies, as well as between puppies and adults, was studied to investigate both self-handicapping and offensive behaviors to determine the extent to which wolves engage in play styles where one individual does not dominate the play. Our results did not support the hypothesized ‘50:50’ rule, which suggests that more advantaged individuals should show higher rates of self-handicapping behaviors in order to facilitate play with others. Adult wolves performed significantly less self-handicapping behaviors than their puppy partners, and they performed significantly more offensive behaviors than their puppy partners. While the ‘50:50’ rule was not supported at any time during our study period, dyads consisting of two puppies had significantly more equal play than dyads consisting of one puppy and one adult. These results suggest that wolf puppies are more likely to play on equal terms with similarly-aged play partners, while the dominance status of the partners dictates offensive and self-handicapping behaviors between animals of different ages.

## Introduction

Inequity aversion refers to when an individual responds negatively when its conspecific partner receives a more highly valued compensation compared with what it has received for the same action [1]. It has been hypothesized that this response has evolved alongside cooperation, as an individual’s ability to compare its rewards to its partner’s would be important in order to recognize a disadvantageous distribution. From this information, the individual could determine whether the partner is worth cooperating with or not [2].

The majority of inequity aversion studies, which have been carried out with primates, have utilized an experimental set-up in which two individuals must perform a similar behavior for an experimenter to obtain a reward, such as an exchange paradigm [3]. The experimenter’s role in some of these tasks has been questioned, as the inequity response may be a product of the experimental setting and the expectations the animals have in relation to the experimenter rather than to the partner [4–6]. Considering these issues, investigating inequity aversion in situations in which there is no interference by the experimenter, and potentially in a natural setting, would be important.

Inequity aversion may be relevant to social play [7,8]. Social play can be viewed as a competitive interaction between play mates, where individuals test and improve their competitive abilities in a ‘safe’ context [9]. However, social play has also been suggested to be a cooperative interaction between two individuals and if this is the case, for play to continue, neither of the playmates should be consistently at a disadvantage [10]. According to this hypothesis, individuals should alternate roles within play (i.e. for example, follow a ‘50:50’ rule [11]), so that both playing individuals show ‘winning’ behaviors, such as chasing or play biting, equally often. It has been suggested that behaviors such as ‘role-reversing’ and so-called ‘self-handicapping’ can be used to maintain equality and thus continue social play [12]. A ‘role reversal’ is defined as when an individual which is dominant to its partner outside of the play context displays a behavior during play that it would not normally adopt in an agonistic interaction, such as muzzle-licking in dogs [12]. In contrast, the term ‘self-handicapping’ does not necessarily take the relative ranks of the partners outside of the play context into account but rather refers to when an individual displays a behavior that might put it into a disadvantageous position during play, such as displaying a belly-up posture whilst lying on the ground, or rolling over [10]. There are three types of self-handicapping: social self-handicapping, when a stronger partner takes a disadvantageous position, kinematic self-handicapping, when a partner exhibits a physically demanding position, and sensory self-handicapping, when a partner closes its eyes while acting [13]. However, for the purposes of this study, we will discuss self-handicapping only in terms of *social* self-handicapping.

The role of equity in play has been investigated only in a few mammalian species, and most studies have investigated whether animals change their behavior within the play context, rather than testing for equity. In support of the hypothesis of equity in play, Biben [14] showed that adult male squirrel monkeys, *Saimiri sqiureus*, would play less aggressively and allow more role-reversals with female play partners to whom they were always dominant, potentially to encourage them to play. Moreover, a small group of adult hamadyras baboons, *Papio hamadryas hamadryas*, were tested for the presence of self-handicapping during play. Results showed that within a pair of play partners, the older individual displayed higher levels of self-handicapping when playing in close proximity to its partner’s more powerful allies [15]. In this case, the baboons’ behavior could also be interpreted as submissive behaviors rather than self-handicapping ones, as the older individuals could be better at taking into account when their partner had allies around. As for non-primate species, captive male red-necked wallabies, *Macropus rufogriseus banksianus*, exhibit higher levels of self-handicapping behaviors towards younger partners, which then allows their partners to gain the advantage during play and take the ‘winning’ position [16]. Bekoff [17] also suggests that individuals which do not play ‘fairly’ may be excluded from play groups. He observed that in coyote puppies, individuals are less likely to engage in play with others who do not play on an even playing field [18].

A number of other studies have found little support for animals engaging in self-handicapping or role reversals to achieve equity in play. A study on play in juvenile gorillas found that individuals which gained an advantage over their partners by hitting them were the first to run away, possibly to keep their partner from hitting back [19]. A study by Pellis and Pellis [20] on Visayan warty pigs (*Sus cebifrons*) found that this species does not appear to show restraint during play, as when one animal gained the advantage on their partner they were likely to attack. However, after this attack, the disadvantaged partners were able to launch a counter-attack approximately 30% of the time. One of several dog studies paired a medium-sized female dog with multiple partners to look at whether or not roll-over behavior within play is a submissive signal [21]. Smaller dogs did not roll onto their backs more, and rolling over behavior was typically categorized as ‘defensive’ to avoid attacks or ‘offensive’ to launch attacks, rather than submissive. However, since the dyads in this study did not know each other beforehand and thus had no relationship with each other, it is still an open question whether or not the dominance relationship between partners affects the exhibition of such behaviors. Bauer and Smuts [8] found that during play between adult dogs all frequenting the same dog park, the dogs did not adhere to the ‘50:50’ rule and instead showed varying degrees of asymmetry in their play. For example, older and more dominant dogs engaged in higher levels of offensive (= winning) behaviors and lower levels of self-handicapping behaviors. Individuals which had an advantage during play, namely through their dominance rank or their age, did not give up their advantage to facilitate play with their disadvantaged partners. Based on these findings it seems behaviors that are closely linked to the expression of dominance and submission outside of the play context continue to be exhibited in accordance to rank also within play. A study on dog puppies found similar results, with puppies not following the ‘50:50’ rule within play and the degree of asymmetry increasing with age [7].

Wolves are a highly social species, exhibiting a strong reliance on cooperation for both breeding and hunting as well as territory and kill defense [22–24]. This, paired with the fact that wolves engage in social play both as puppies and as adults, within and between age groups, suggests they may be a particularly good species to study equity within the play context. However, only a few studies on wolf play behavior have been carried out, with most of these studies focusing on adult animals, and none looking specifically at inequity aversion or equality within the play context [25–29]. In the current study, we investigate play behavior in wolves as puppies in relation to both the ‘50:50’ rule, and in relation to their dominance relationship outside of play.

Looking at previous studies conducted with dogs [7,8] and Cordoni’s [26] study in wolves, we could hypothesize that wolves will not follow the ‘50:50’ rule, but will maintain the same dominance relationship evidenced outside of play also within the play context. However, due to the differing dependence of wolves and dogs on cooperative activities [30], we may expect differences in their propensity for equal play. While wolves are highly reliant on other pack members [22,24], dogs show a much reduced pack involvement in pup-raising [31] and their foraging strategies show a greater reliance on scavenging than group hunting (e.g., scavenging at rubbish dumps, or food provisioned by humans; [32–34], but see also [34]). Accordingly, play behavior in wolves may show a greater symmetry than in dogs.

To test whether wolves attend to equality by following the ‘50:50’ rule, we calculated a win ratio for each dyad. The win ratio was calculated in terms of the number of times an individual was seen performing an offensive behavior (= being in the winning position), versus the number of times an individual was seen performing a self-handicapping behavior (= being in the losing position). Specifically, we did not calculate a winner or a loser for the interaction, but rather, how often either individual was seen in a position, which could be termed winning or losing. These terms are often used in reference to offensive and self-handicapping behaviours, respectively [7,8]. Offensive behaviors were defined as those used by individuals to gain a competitive advantage, such as biting and chasing their partner. Self-handicapping behaviors were defined as those used by individuals to give up a competitive advantage to their partner, such as laying on the back as well as being physically under their partner. We analyzed how the age of the play partner (both puppies or one puppy and one adult) influenced play behavior, but also how pack composition (puppy or mixed-age pack) influenced play behavior within the puppy dyads. Furthermore, based on general observations of the interactions between individuals, we measured their rank relationships outside of play and analyzed whether this affected the occurrence of offensive and self-handicapping behaviors within the play context. With these general observations, we calculated a win ratio for each dyad outside of play in order to compare it to the win ratio for those dyads inside of play. To test the hypothesis that animals adhere to the ‘50:50’ rule, we tested a number of predictions in relation to the ratio of offensive and self-handicapping behaviors within play and the means by which this ratio might be achieved during play.

### 50:50 Ratio

If, in accordance with the ‘50:50 ratio’ hypothesis [10,11], wolves play equitably, then the win ratio of most dyads should be situated around ‘50:50’ in the play context. There should also be no correlation between the dyads’ win ratios inside of play and their win ratios outside of play. However, if wolves do not adhere to the ‘50:50’ rule, but maintain their relationships from outside of play also within the play context, we should find varying levels of asymmetry in the win ratios of the dyads as was found in dogs [7,8] and they should be correlated with relationship status from outside of play. According to Bekoff [17], individuals which do not play symmetrically with partners may be avoided in the play contexts. On this basis, dyads which have less equitable play should engage in less play than dyads which have more equitable play.

### Offensive and Self-Handicapping Behaviors within Play

According to some researchers [11,12], more advantaged individuals should engage in more self-handicapping within play than would be expected in order to keep the play more symmetrical. Thus, based on this hypothesis, the dominant individual within a pair (as established outside the play context) should engage in as many self-handicapping behaviors as the subordinate individual. Alternatively, if wolves do not attempt to maintain equity within play, more advantaged individuals should continue to push their advantage also during play. Therefore, dominant individuals should engage in more offensive behaviors and subordinate individuals in more self-handicapping behaviors within play.

Furthermore, in mixed age dyads, if advantaged individuals do exhibit these self-handicapping behaviors to keep play more symmetrical, we would expect these behaviors more in adults when they are playing with puppies, due to their advantage over the puppies outside of the play context. Alternatively, if wolves do not follow the hypothesis on equity in play, we would expect to see older individuals engaging in higher rates of offensive behaviors, with their disadvantaged partners engaging in higher levels of self-handicapping behaviors. In summary, we predicted that if play between wolf puppies followed the pattern of play between dog puppies, then wolves would not follow the ‘50:50’ rule and the subsequent patterns of offensive and self-handicapping behaviors would show no evidence of equity in the play. However, if the differing dependence of wolves and dogs on cooperation has affected the play behaviors of wolves, then we may find that wolves do follow the ‘50:50’ rule and the subsequent patterns of offensive and self-handicapping behaviors would show evidence of equity in the play.

## Methods

### Ethical Statement

No special permission for use of animals (wolves) in such socio-cognitive studies is required in Austria (Tierversuchsgesetz 2012– TVG 2012). The relevant committee that allows running research without special permissions regarding animals is: Tierversuchs-kommission am Bundesministerium für Wissenschaft und Forschung (Austria).

### Subjects

Two packs of wolf (*Canis lupus occidentalis*) puppies aged three to five months were observed for play interactions as well as non-play interactions at the Wolf Science Center (WSC) in Ernstbrunn, Austria in 2009 and 2012 respectively. Each pack consisted of six wolf puppies with both kin and non-kin individuals but with no more than two individuals from one litter per pack (see Table 1). Wolves were brought to the center by ten days of age and were hand-raised by professional trainers, and thus spent all of their time in the presence of a human from the age of ten days to four months (for more information on raising methods, see [35,36]). During these first four months the puppies lived in a single pack in an enclosure; afterwards, individuals were introduced into previously established packs of adult wolves (into mixed-age packs). In 2009, the six puppies (4 males and 2 females) were integrated into a previously established group of three adult wolves (2 males and 1 female) from the 2008 litter, and this pack of nine individuals was analyzed for play after introduction. In 2012, the six wolf puppies (3 males and 3 females) were separated into three different, previously-established packs of adults from the 2009 pack as well as from a separate, 2010 litter. To see the details of the packs observed for each study period, as well as information in related individuals, see Table 1. Filming for both play and non-play interactions took place in the animal’s home enclosures, which consisted of large, fenced outdoor areas, raised platforms for shelters, as well many trees and sometimes fallen tree trunks.

**Table 1:** Distribution of subjects within the packs for both study periods.

Subject	Sex	2009 Puppy Pack	2009 Mixed-Age Pack	2012 Puppy pack	2012 Mixed-Age packs
Kaspar	M	—	Kaspar, 2009	—	Kaspar, 2012
Shima^a^	F	—	Kaspar, 2009	—	Kaspar, 2012
Aragorn^a^	M	—	Kaspar, 2009	—	Kaspar, 2012
Tala^b^	F	—	—	Puppy Pack	Kaspar, 2012
Chitto^c^	M	—	—	Puppy Pack	Kaspar, 2012
Nanuk	M	Puppy Pack	Kaspar, 2009	—	Nanuk, 2012
Yukon^d^	F	Puppy Pack	Kaspar, 2009	—	Nanuk, 2012
Una^c^	F	—	—	Puppy Pack	Nanuk, 2012
Wambleee	M	—	—	Puppy Pack	Nanuk, 2012
Geronimo^d^	M	Puppy Pack	Kaspar, 2009	—	Geronimo, 2012
Kenai	M	—	—	—	Geronimo, 2012
Amarok^b^	M	—	—	Puppy Pack	Geronimo, 2012
Kay^e^	F	—	—	Puppy Pack	Geronimo, 2012
Apache^f^	M	Puppy Pack	Kaspar, 2009	—	DECEASED
Cherokee^f^	M	Puppy Pack	Kaspar, 2009	—	DECEASED
Tatonga	F	Puppy Pack	Kaspar, 2009	—	REMOVED

Packs other than puppy packs are named by ‘Alpha Male, Year.’

^a-f^ matching letters denote siblings

### Data Collection

All interactions were recorded with a video camera (Handycam DCR-SR35 Sony) for later coding. For the 2009 collection period, all observations were taken from the beginning of August 2009 until the end of November 2009. For the 2012 collection period, all observations were taken from the beginning of September 2012 until the end of November 2012. All observations were distributed throughout the day, from approximately 0600 to 2000 hours. Two types of data were collected for the purpose of the study: play observations, which were used to code play behaviors, and general observations, which were used to code non-play behaviors.

We defined play by indicators as suggested by recent studies on play in canids [7,8,26]; these include the exaggerated, repetitive, and fragmented behaviors in Table 2 which when seen together were used as our working definition of a play bout. A play session began when one partner directed any playful behaviour towards its play partner, and ended when the participants stopped their behaviors or one moved away [37]. Instances in which one animal directed playful behaviors to a partner who ignored them were not considered play and were not included in the analyses. Videos used for this study were recorded in the framework of an earlier study by Heufelder [38] and re-coded for the purposes of this study. Recordings were started when the focal animal started to play or was already playing. One focal animal was filmed continuously for 5 minutes. Focal animals were chosen after the play had started, rather than before, and were chosen pseudo-randomly in an attempt to keep an evenly distributed amount of focal videos for each individual. After 5 minutes filming ended, regardless of whether or not the play session had ended. While every occurrence of play was not recorded during the observation time, all occurrences of play on the videos, even if it did not include the original focal individual, were coded and used in the present analyses. For the study period 2009, 8.1 hours of play videos were recorded of the puppy pack, and 5.7 hours of play videos were recorded in the mixed-age pack. For the study period 2012, 7.5 hours of play videos were recorded of the puppies in the puppy pack, and 5.9 hours of play videos were recorded in the mixed-age packs.

**Table 2:** Ethogram of behaviors coded in the play context, adapted from previous studies [7,8,26].

Behavior	Definition
**Offensive Behaviors**	
Bite shake	Actor (A) * bites recipient (R)** and shakes head back and forth while maintaining a hold on R
Play bite	A gives an inhibited bite to R (without shaking the head)
Chase/charge	A runs after R with a least two running strides while R runs or trots away from A, or A breaks from a stalking position into a run, moving directly towards R.
Chin over	A places the underside of chin over R’s back, usually right behind the neck or near R’s shoulders, but sometimes over R’s head.
Paw on	A stands up on its hind legs and puts front legs on R’s shoulders, usually silent and with open mouth, individuals can bite each other.
Forced down	A uses physical force or contact to cause R to drop completely to the ground from a moving, standing or sitting position. Force may be applied with a bite (pin), push/tackle, body slam, bouncing into him (knock down) or some other forceful movement.
Mount (push/tackle)	A rears up (keeping hind legs on the ground) to place forelegs on R’s back. A has a rounded spine with curved front legs and forepaws to grasp R’s torso. Pelvic thrusting may or may not be present (if it results in a down, it was coded as forced down instead of independent push/tackle).
Muzzle bite	A places mouth around R’s muzzle
Over	A sits on, stands over, or lies over R with at least 25% of A’s torso over R’s torso
Overs during downs	A stands over or lies on R with at least 50% of A’s torso over R’s torso (or vice versa: 50% of A’s torso is under R’s torso), or A sits and exerts weight directly on R’s head or torso with a distinct pause in the sitting position
**Self-Handicapping Behaviors**	
Muzzle lick	A licks on or around R’s muzzle. A lick may or may not be accompanied by nudging
Receive genital sniff	A holds hind legs apart while in belly-up position on the ground to allow R to put snout on or near A’s genitals for an investigatory sniff
Voluntary down	A drops completely to the ground from a moving, standing or sitting position without R’s physical enforcement. R and A must be interacting when A goes down
Unknown down	Definition same as ‘voluntary down,’ however, owing to the camera angle, it is unclear whether the down is forced or voluntary, but a definite asymmetry in positions exists

* A: wolf is performing the behavior

** R: wolf is receiving the behavior

General observations were taken in study period 2012 to determine dominance relationships between individuals outside of play. These were taken in the form of video recording that we subsequently coded. 14.2 hours of general observations were recorded in the puppy pack, and 17.7 hours of general observations were recorded in the mixed-age packs. For each video, one individual was followed for ten minutes, however, non-play behaviors were coded for all individuals, not just the focal individual. This allowed us to increase our number of non-play interactions in the analysis of the dominance hierarchy. For study period 2009, we had access to ‘live’ general observations done on just the focal individual by Stefanie Heufelder [38] using the program Pocket Observer Version 2.1.23.2 (Noldus Information Technology) on an HP iPAQ (Hewlett-Packard) pocket computer. However, due to the low number of interactions from these live focal observations, we integrated into our analyses data for both the puppy pack for 2009 as well as the mixed-age pack for 2009 from videos taken for a separate study in a feeding context (unpublished data). In both wolves and dogs there is evidence that dominance relationships observed in the social context are mirrored also within the feeding context [39], allowing us to use material from both contexts to calculate the dominance relationships amongst individuals.

We used submissive and dominance interactions but not aggressive interactions to determine dominance relationships since aggressive interactions are often not unidirectional. The behaviors used to code our general observations are summarized in Table 3. We analyzed the data for each observation period (puppy pack and mixed-age pack) separately, to account for possible changes in the dominance relationships over time.

**Table 3:** Ethogram used to determine the rank relationships outside of the play context. Adapted from the Wolf Science Center Social Behavior Ethogram.

Behavior	Definition
**Dominant Behaviors**	
Stand Tall	A* straightens up to full height, with a rigid posture and tail, may include raised hackles, ears erect and tail perpendicular or above the back.
Stand Over	A is standing over R’s** body, with all four paws on the ground, with the tail held high. R may have either the whole body or just the forepaws under A’s belly/side.
No-Play Paw On	A places or both forepaws on the R’s back—outside of the play context.
Ride Up	A mounts R from behind or from the side, exhibiting a thrusting motion.
Head On	A approaches R’s shoulder/back and puts its head on it. Most of times formation looks like a capital “T”.
No-Play Muzzle Bite	A grabs the muzzle of R either softly or with enough pressure to make the other whimper—outside of the play context.
**Submissive Behaviors**	
Crouch	A lowers the head, sometimes bending the legs, arching the back, lowering the tail between the hindlegs, and avoiding eye contact.
Passive Submission	A lies on the back showing the stomach and holding the tail between the legs. The ears are held back and close to the head and the subject raises a hind leg for inguinal presentation.
Active Submission	A has its tail tucked between the hind legs sometimes wagging it while he is in a crouched position (with hindquarters lowered) and may attempt to paw and lick the side of R’s muzzle. The behavior may include urination.
Withdrawing	A withdraws from R moving away slowly in the opposite direction, displaying a submissive posture. It occurs when an A has been threatened or attacked by R, or a fight has taken place.
Flee	A runs away from R with tail tucked between the legs and body ducked. It occurs when an A has been threatened or attacked by R, or after a fight.
Avoidance	In response to R reducing the distance towards it, the A moves away displaying a submissive posture. The A may also look at the individual he is trying to avoid.

* A: wolf is performing the behavior

** R: wolf is receiving the behavior

### Analysis

Video recordings of the play sessions and the general observations were coded in the program Observer XT 9.0 (Noldus Information Technologies).

#### General Observations

Rank relationships (i.e. who was dominant and who submissive in each dyad), for all packs/periods, were determined by adding dominance interactions to the reversed score for submissive interactions. For each pack/period the individual frequencies of these behavioral categories were ranked in a matrix with actors in rows and receivers in columns. For each matrix, the linearity (1 = completely linear) and unidirectionality (1 = completely unidirectional) were calculated following the procedures proposed by de Vries [40] and van Hooff and Wensing [41], respectively. The level of unidirectionality refers to the frequency with which in each dyad a specific behavior is exhibited consistently from one individual to the other. At the group level the higher the level of unidirectionality the clearer the dominance hierarchy emerging. These matrices were then organized so as to reduce the number of inconsistencies, which are defined relatively to the current rank order as a lower-ranking individual that dominates a higher ranking individual [40]. These analyses were performed using the program Matman 1.1 (Noldus Information Technology, Wageningen, The Netherlands). The rank relationships, i.e. dominant and subordinate individual in each dyad, were then used to assess the effect of the dominance relationship on the occurrence of offensive and self-handicapping behaviors during play. Individuals were reordered in the matrices according to their ranking position (see S7–S12 Tables). However, within the analyses, the dominance relationship was entered for each individual as whether they were “dominant” or “submissive” to their partners, rather than using a ranking position or score to calculate the distance between them.

#### Play Observations

The ethogram includes definitions from Ward et al. [7], Bauer and Smuts [8], Cordoni [26], and Heufelder [38]. The ethogram was separated into different types of interactions: offensive behavioral patterns and self-handicapping behavioral patterns. For definitions see Table 2. Dyadic play was only coded when both individuals were engaged in play, and not when one individual was merely persistent in play invitations. Due to the fact that we did not always have the beginning of the play bout within the videos, play invitations were not used to analyze any playmate preferences, as we could not determine necessarily whether these were play invitations entirely outside of the play context (e.g. if the individuals had been playing at some point just before the recording, but stopped). Play bouts were ended and coded as a second play bout if individuals stopped playing for 15 seconds or more. To ensure that we did not skew the data with dyads which played only for a few seconds over the course of the entire study period, we only included dyads which had at least one minute of total play time (all bouts added). All videos were coded by J.E. and S.C., except videos for the 2009 general observation data, which were coded by Teresa Schmidjell. Interobserver reliability for the play observations based on 20% of the videos was high (all behaviors above κ = 0.75).

#### Testing the ‘50:50’ Ratio

To test for the symmetry of play, as well as whether or not advantaged individuals from outside of play facilitate play with less-advantaged partners, individuals were said to be in the “winning” or “losing” positions depending on the behaviors they were exhibiting. Inside of play, offensive behaviors were considered “winning,” while self-handicapping behaviors were considered “losing” (for definitions of offensive and self-handicapping behaviors, see Table 2). Offensive and self-handicapping behaviors have been termed “winning” and “losing” behaviors, respectively, in other studies on play in canids [7,8]. In this way, we could define a ratio for each pair of how often one was in the winning or losing position depending on the number of times each individual exhibited offensive or self-handicapping behaviors within the play bouts. Outside of play, dominant behaviors were considered “winning,” while submissive behaviors were considered “losing” (see Table 3).

$$Winning\;Index=\;\frac{\left(A_{offensive}+B_{self-handicapping}\right)-\left(B_{offensive}+A_{self-handicapping}\right)}{(A_{offensive}+B_{self-handicapping})+\left(B_{offensive}+A_{self-handicapping}\right)}$$

**Eq 1**—Winning index based on offensive and self-handicapping behaviors performed by Subject A and Subject B during play.

The proportion could range from 50:50 (complete symmetry) to 100:0 (complete asymmetry).

This ratio was calculated using the frequencies of each offensive and submissive behavior. We used a Wilcoxon Signed-Ranks Test to compare the win ratios outside and inside of play, and a Wilcoxon Test to test for symmetry within play. These analyses were conducted in SPSS v20.0.0.

#### Test Models

To further test if individuals engaged in behaviors achieving a 50:50 ratio, we used linear mixed models (LMMs) and generalized linear mixed models corrected for over-dispersion when necessary (GLMMs). As individuals were parts of multiple dyads, and each dyad could occur more than once if they were both part of the same puppy and mixed-age packs, we included individual and pair as a random factor to avoid pseudoreplication. We also included play duration for each dyad as a control variable in models where applicable, to control for dyads which varied in their overall duration of play time. All model analyses were conducted in R v3.1.2. We used a model reduction method based on p-values. Variables employed for each model can be seen in S1–S6 Tables.

#### Model 1: What affects the win ratio in play for puppy dyads?

Considering only dyads in which both individuals were puppies, we ran a linear mixed model (LMM) with the win ratios as the response variable and ‘pack type’ (to determine whether the response variable is affected by whether dyads were in a puppy or a mixed-age pack), ‘sex’ and ‘play duration’ as predictor variables.

#### Models 2 and 3: Does the dominance relationship within puppy dyads affect the frequency of self-handicapping (Model 2) and offensive (Model 3) behaviors exhibited in play?

Considering only dyads in which both individuals were puppies, we ran a GLMM with a Poisson distribution, with the frequency of either self-handicapping or offensive behaviors as the response variable and ‘dominance relationship’ to partner (i.e. subordinate vs. dominant) and the interaction between ‘pack type’ and ‘dominance relationship’ to partner (to control for the possibility that rank relationships affect the response variable differently if puppies are in the puppy or mixed-age packs) as predictor variables.

#### Model 4: Is play between mixed-age dyads more equal than play between puppy-puppy dyads?

Considering only data from mixed aged packs, we ran a LMM with the win ratios as the response variable, and ‘dyad type’ (i.e. either ‘AP’ adult- puppy or ‘PP’ puppy-puppy), ‘sex’ and ‘play duration’ as predictor variables.

#### Models 5 and 6: Do adult wolves show more self-handicapping (Model 5) and less offensive behaviors (Model 6) than expected in their play with puppies in order to facilitate play?

Considering only data from mixed aged packs, we ran a GLMM with a Poisson distribution, with either the frequency of self-handicapping or offensive behaviors as the response variable and ‘dyad type’ (i.e. ‘AP’ adult-puppy or ‘PP’ puppy-puppy), ‘dominance relationship’ to partner (subordinate vs. dominant) and ‘sex’ as predictor variables. To determine whether the subject’s dominance relationship to their partner would affect the frequency of self-handicapping or offensive behaviors in play differently depending on dyad type, we included the interaction between these two predictor variables.

## Results

### General Observations: Dominance Relationships

For the mixed-age packs, both the linearity and unidirectionality were high, and where the number of subjects allowed for statistical testing (N≥6, i.e. Kaspar 2009), they proved to be significant (see Table 4). In both puppy packs, we have one inconsistency and a lower unidirectionality than in the adult packs, which had no inconsistencies (see Table 4). In the mixed-age packs, adults were always dominant to puppies (see S7–S12 Tables).

**Table 4:** Summary of the values of the two main properties of the behavioral category analyzed for each pack: the improved linearity index (h') and the directional consistency index (DCI).

Pack	Pack size	h'^a^	DCI^b^	Right-Tailed Probability
Puppy 2009	6	0.771	0.523	0.12
Kaspar 2009	9	0.93	0.91	<0.01
Puppy 2012	6	0.8	0.659	0.05
Geronimo 2012	4	1	0.92	—
Nanuk 2012	4	0.9	0.929	—
Kaspar 2012	5	0.95	0.971	—

^a^ (de Vries, 1995)

^b^ (Van Hooff and Wensing, 1987)

### ‘50:50’ Rule

**Fig 1:**
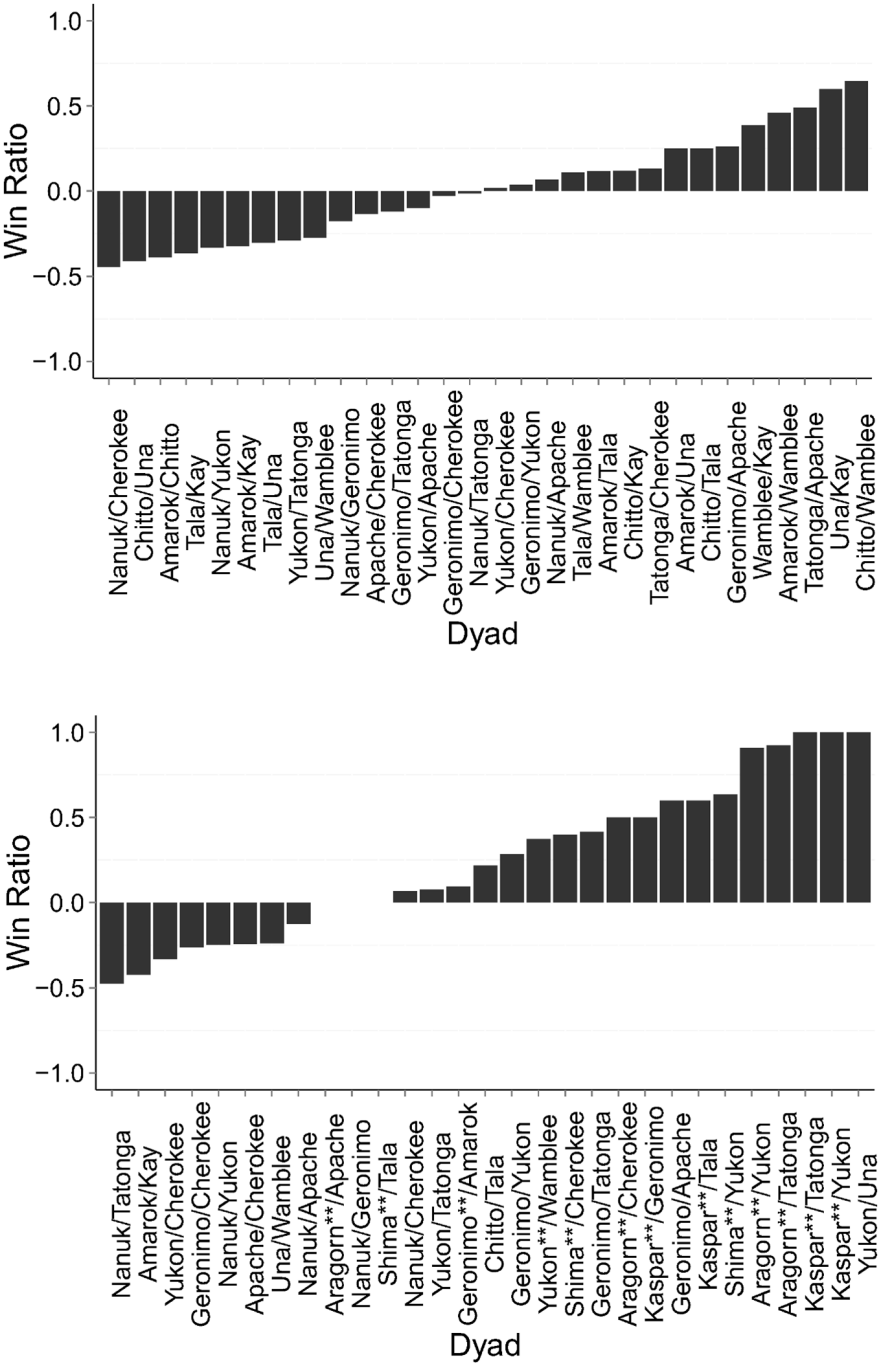
(a and b). Win Ratios for each Pack Type. A win ratio of "0" would represent complete symmetry, with each partner being in the 'winning' position an equal amount of times. A win ratio of '1' or ‘-1’ is complete asymmetry, with one individual being in the ‘winning’ position the entire time the dyad was seen playing. Dyad names are listed arbitrarily, however a win ratio of less than ‘0’ represents a dyad where the win ratio is skewed towards the individual who is subordinate outside of play being in the ‘winning’ position most often inside of play. Fig 1a (above) represents dyads in the puppy packs, and Fig 1b (below) represents dyads in the mixed-age packs. Adult wolves are denoted with ** after their name.

We found that the ‘50:50’ rule was not supported in our wolves, since the win ratio during play deviated significantly from an equal distribution in the puppy packs (Mean = 0.007, One-Sample Wilcoxon Signed-Ranks: z = 4.780, p<0.001, n = 30) as well as in the mixed-age packs (Mean = 0.25, One-Sample Wilcoxon Signed-Ranks: z = 4.458, p<0.001, n = 29) (See Fig 1). However, for each dyad, win ratios inside of play were significantly different from win ratios outside of play for the puppy packs (Two-Sample Wilcoxon Signed-Ranks: z = -2.573, p = 0.01, n = 30) as well as for the mixed-age packs (Two-Sample Wilcoxon Signed-Ranks: z = -2.763, p = 0.006, n = 29).

### Puppy-Puppy dyads

#### Model 1. What affects the win ratio in play for puppy dyads?

In regards to the win ratios for the puppy dyads, we found no significant effects of the ‘pack type’ (Puppy pack Mean = 0.007, Mixed-age pack Mean = 0.000, LMM: F_1_ = 0.068, p = 0.794), hence wolf puppies did not play more equally at a younger age, nor did the presence of adult wolves affect the win ratios of the puppy dyads. No effect of ‘sex’ (LMM: F_2_ = 0.920, p = 0.631) and no effect of ‘play duration’ (LMM: F_1_ = 0.331, p = 0.565) were found, the latter suggesting that ‘equal’ play does not correlate with more play between wolf puppy dyads.

#### Model 2 & 3. What affects the frequency of self-handicapping and offensive behaviors in play for puppy dyads?

We found no effect of the ‘dominance relationship’ on the frequency of self-handicapping or offensive interactions for the puppy dyads (GLMM: F_1_ = 0.001, p = 0.974; F_1_ = 0.022, p = 0.882). Also, as there was no significant interaction between ‘dominance relationship’ and ‘pack type’ (GLMM: F_1_ = 0.416, p = 0.519; F_1_ = 0.021, p = 0.885), this was the case for puppy dyads in the puppy packs as well as in the mixed-age packs. Thus, regardless of whether or not puppies were in a puppy pack or a mixed-age pack, the dominance relationship between the pairs had no effect on the frequency of self-handicapping or offensive behaviors inside of play. There was also no effect of ‘sex’ (GLMM: F_2_ = 1.029, p = 0.598; F_2_ = 3.591, p = 0.166). However, puppies engaged in more self-handicapping and offensive behaviors at a younger age when they were in the puppy pack compared to at an older age when they were in the mixed-age pack (GLMM: F_1_ = 18.201, p<0.001; F_1_ = 7.6898, p = 0.006).

### Mixed-Age Packs

#### Model 4. What affects the win ratio in play in mixed-age packs?

**Fig 2:**
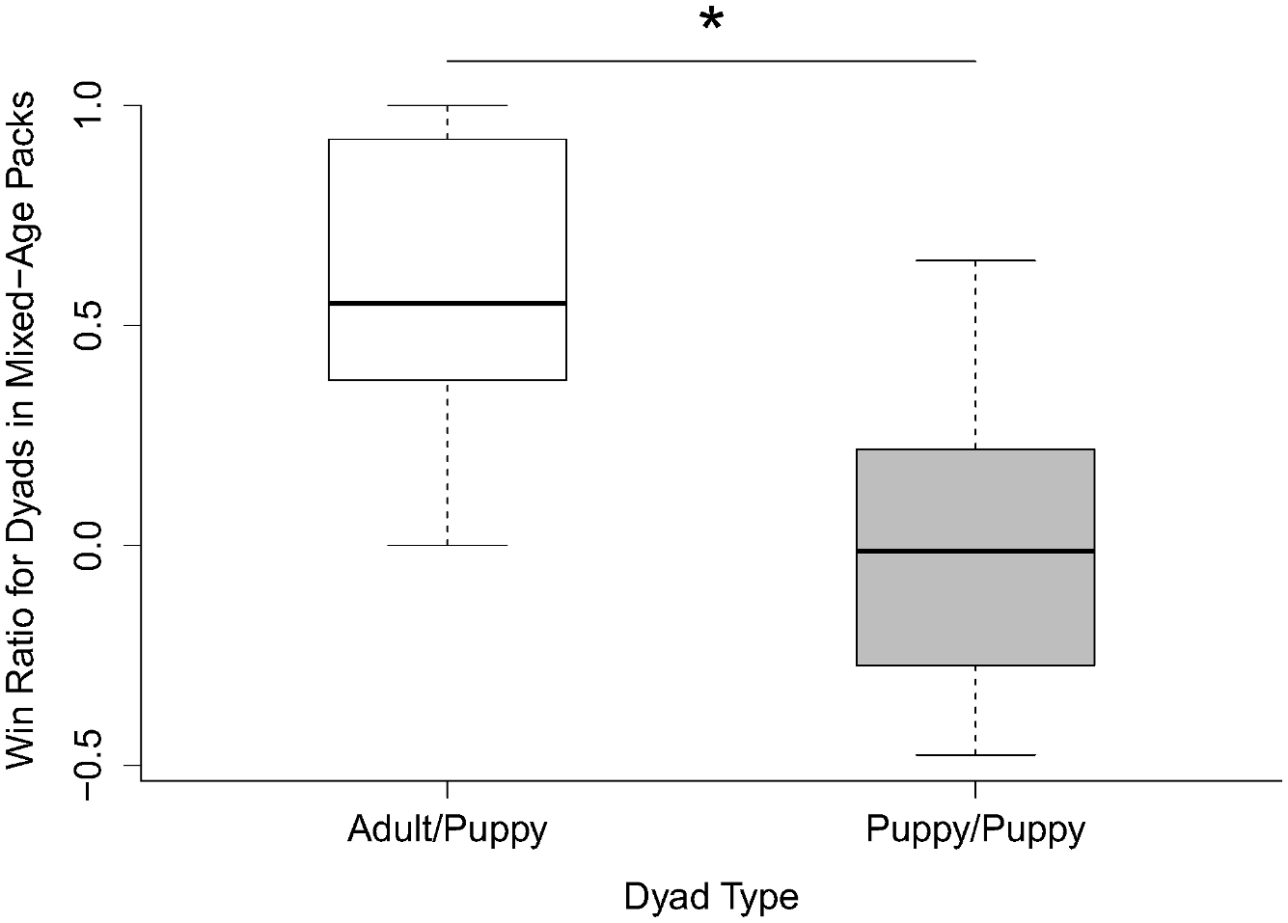
Win ratio for the adult/puppy and puppy/puppy pairs in the mixed-age packs. * p<0.01.

In regards to the win ratios for the mixed-age packs, we found a significant effect of the dyad type on the win ratio (LM: F_1_ = 8.20, p = 0.008). The dyads with two puppies (Mean = -0.046) were significantly closer to ‘equal’ play than the dyads with an adult and a puppy (Mean = 0.567) (Fig 2). We also found a tendency for ‘sex’ to have an effect on the win ratio (LM: F_1_ = 3.231, p = 0.056) with male-female dyads exhibiting the least symmetrical play, and male-male dyads exhibiting the most symmetrical play. We found no effect of the ‘play duration’ (LM: F_1_ = 0.291, p = 0.595), suggesting ‘equal’ play does not correlate with more play also in the mixed-age packs.

#### Model 5. What affects the frequency of self-handicapping behaviors in play in mixed-age packs?

**Fig 3:**
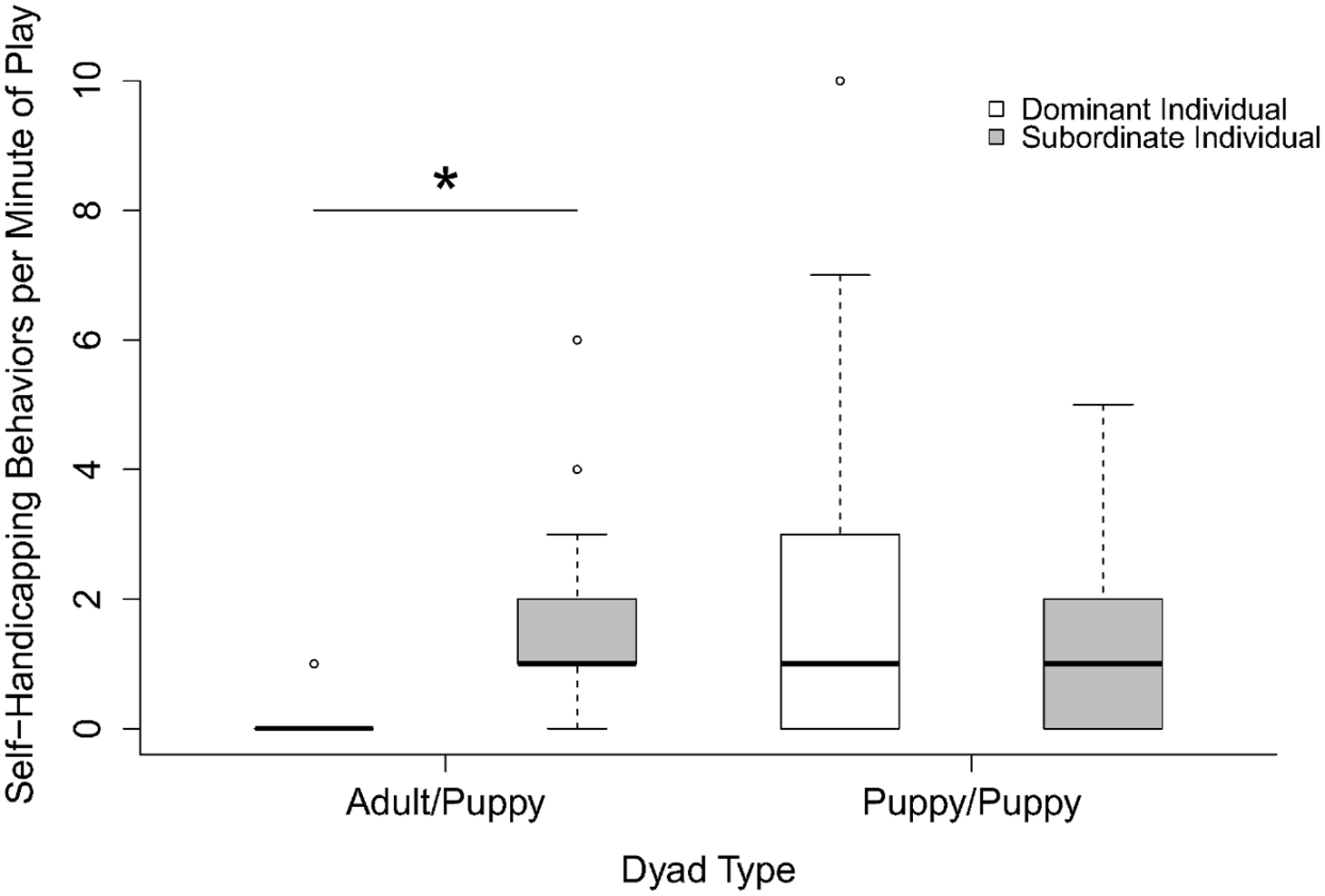
Frequency of self-handicapping behaviors by individuals in adult/puppy and puppy/puppy dyads in the mixed-age packs. * p<0.001.

In regards to the self-handicapping behaviors in play for dyads in mixed-age packs, we found a significant interaction between ‘dominance relationship’ to partner and the ‘dyad type’ (GLMM: F_1_ = 13.581, p<0.001). In the dyads with an adult and a puppy, the subordinate/younger individual performed significantly more self-handicapping behaviors than the dominant/older individual (GLMM: F_1_ = 5.312, p = 0.021: Fig 3). However, in the dyads with two puppies, we found no significant effect of ‘dominance relationship’ on self-handicapping behaviors in the mixed-age pack (GLMM: F_1_ = 0.181, p = 0.670: Fig 3).

#### Model 6. What affects the frequency of offensive behaviors in play in mixed-age packs?

**Fig 4:**
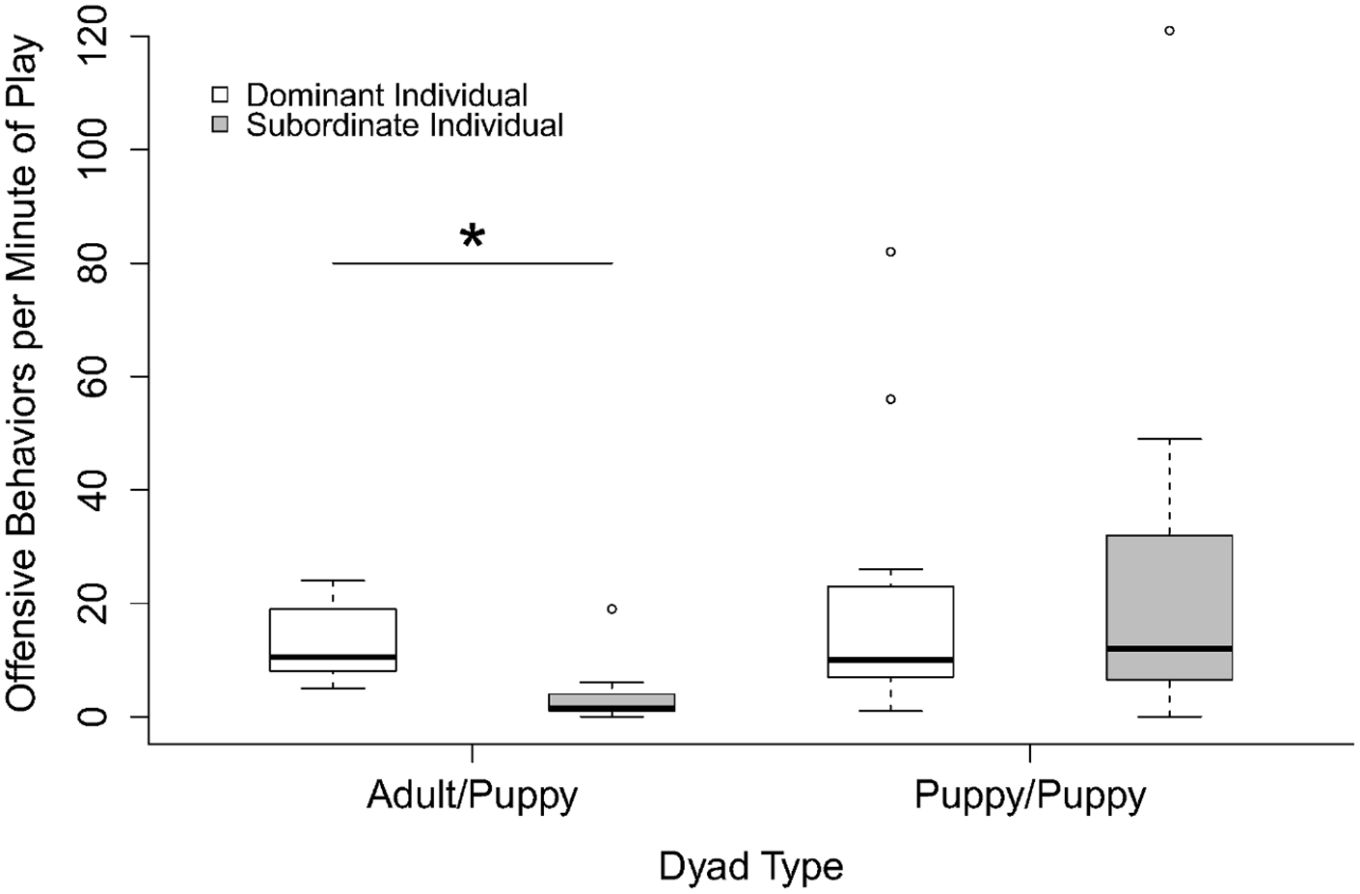
Frequency of offensive behaviors by individuals in adult/puppy and puppy/puppy dyads in the mixed-age packs. * p<0.001.

In regards to the frequency of offensive behaviors in play in mixed-age packs, we again found an interaction between ‘dominance relationship’ to partner and ‘dyad type’ (GLMM: F_1_ = 28.666, p<0.001). In the dyads with an adult and a puppy, the dominant/older individual performed significantly more offensive behaviors than the subordinate/younger individual (GLMM: F_1_ = 31.048, p<0.001: Fig 4). However, in the dyads with two puppies, we found no significant effect of ‘dominance relationship’ (GLMM: F_1_ = 1.025, p = 0.311: Fig 4). We found no effect of ‘sex’ (GLMM: F_2_ = 1.741, p = 0.419)

## Discussion

Wolf puppies did not follow the proposed ‘50:50’ rule during dyadic play, and play ranged from equal to completely unequal within our dyads. However, we found that dyads consisting of two puppies engaged in significantly more equal play than dyads consisting of one adult and one puppy in the mixed-age packs. Moreover, in dyads consisting of two puppies, there was no evidence that the dominance relationship affected either the rates of self-handicapping or offensive behaviors in play. However, in mixed pairs of one adult and one puppy, puppies engaged in significantly more self-handicapping behaviors, and significantly less offensive behaviors than their adult partners. Thus, contrary to predictions based on the 50:50 rule, adult wolves did not facilitate play by exhibiting more self-handicapping and less offensive behaviors towards their partners than would be predicted by their dominance relationship. However, more dominant puppies did not take advantage of their higher position outside of play, engaging in neither more offensive, nor less self-handicapping, behaviors than their lower-ranking puppy partners. This may be due to the fact that the dominance ranks are not fully established yet, nevertheless, the play interactions appear to be more equal than those between adults and puppies. Hence, play between puppies may have a competitive component, with individuals testing their abilities against each other in order to acquire a higher rank in the future [42]. We also found no effect of the win ratio on play duration, so again, contrary to predictions based on one of Bekoff's hypotheses, dyads which had more unequal levels of play did not engage in less play than dyads which exhibited more equal play [18], and this was true for both the puppy packs as well as the mixed-age packs.

The rates of self-handicapping and offensive behaviors in puppy-adult dyads were tied to the dominance relationship between them, supporting the hypothesis that interactions within play are not separate from the dominance relationship outside of play. The subordinate individual performed self-handicapping behaviors within play more frequently than the dominant individual, who performed more offensive behaviors. It is important to reiterate that the dominant individual was invariably the adult individual in the dyad. This means that adult, dominant wolves performed more offensive behaviors, and young, subordinate wolves performed more self-handicapping behaviors. Thus, at least for the mixed-age dyads, the dominant and subordinate roles which are established outside of play are actually maintained within the play context. In contrast to the mixed-age dyads, puppy dyads did play more equally which might reflect the lower unidirectionality in the puppy packs. The reduced unidirectionality in the puppy packs may be due to hierarchical relationships being poorly defined. While we found a high degree of linearity and no circular relationships in the analyses of the mixed-age packs, unidirectionality was much lower in the puppy packs suggesting that the puppy-puppy relationships were, at this point, still quite undefined. This lack of defined dominance relationships, which continued also when the puppies were introduced into the adult packs, could also explain why there was no decrease in symmetry in play as the puppies age (e.g. when they were moved from the puppy packs to the mixed-age packs). While in 2012, all puppies retained their rankings relative to the other puppies once introduced into the mixed-age packs, in 2009, there were some changes in the rank positions of the puppies once introduced into the mixed-age pack (see S7–S12 Tables). Thus, it is possible that the puppy-puppy dominance relationships are in some way affected by the presence of adults, however to what extent the relationships are affected, we cannot speculate with the present data. Pairs of rats show a decrease in symmetry in play as they establish their dominance relationship [43]. Domestic dog puppies decrease their symmetry in play over time when comparing the time periods between 3 to 8 and 10 to 23 weeks of age. Interestingly, however, no such decrease can be detected when comparing the time periods between 10 to 23 weeks of age and 27 to 40 weeks of age [7], which could be due to the establishment of dominance relationships in the later but not earlier weeks. In our study, no such decrease could be observed despite the fact that our animals were 12 to 20 weeks of age, suggesting the possibility that wolf pups establish their dominance relationships later than dogs and hence play remains more symmetrical for longer. However, this hypothesis needs further testing.

It is important to note that there is a large variability in the ‘equity’ with which puppy dyads play, with some playing on quite equal terms (see Fig 1a). Since equity during play is not correlated with the dominance outside of play, other aspects of the relationship, for example affiliation, could affect the play style. In groups of chimpanzees, relationship length, and perhaps relationship quality, does affect inequity aversion [44]. Conversely, in domestic dogs, pairs with a more affiliative relationship showed higher levels of inequity aversion than pairs with a less affiliative relationship [45]. Accordingly, how affiliation affects equity in play needs to be assessed in future studies.

In summary, our study presents the first evidence, to our knowledge, that wolf puppies do not adhere to the ‘50:50’ rule within the play context. However, the ‘50:50’ rule is likely not the only way to test for egalitarian play, and there may be other ways that play can be facilitated between individuals which would need further investigation. Furthermore, adult wolves do not engage in self-handicapping behaviors to facilitate play with puppies. Instead, wolves, like dogs, can and do engage in dyadic play at similar rates regardless of the symmetry of the play between the individuals. Thus, it does not appear that the retained levels of conspecific cooperation within wolves, compared to dogs, has selected for more egalitarian play styles. However, future studies should be done on pack-living domestic dogs in order to be able to make more valid comparisons between the two species.

## Supporting Information

S1 Data Win Ratio Data. (XLSX)

S2 Data Offensive and Self-Handicapping Behavior Data. (XLSX)

S1 Table Outputs from the Model 1 analysis. Linear mixed effects model with the win ratios of the puppy-puppy dyads as the response variable with ‘pack type’ (e.g. ‘puppy pack’ versus ‘mixed-age pack’), ‘sex mix’ of the dyad, and the ‘play duration’ for the dyad as the predictor variables. Statistics are given for each variable when they were last in the model.
(DOCX)

S2 Table Outputs from the Model 2 analysis. Generalized linear mixed effects model with the frequency of self-handicapping behaviors of the puppy-puppy dyads as the response variable. ‘Relation’ of the individual to its dyadic partner (e.g. whether they were ‘subordinate’ or ‘dominant’), ‘sex mix’ of the dyad, and the ‘pack type’ for the dyad (e.g. ‘puppy pack’ versus ‘mixed-age pack’) were predictor variables. An interaction between ‘relation’ and ‘pack type’ was included. Statistics are given for each variable when they were last in the model.
(DOCX)

S3 Table Outputs from the Model 3 analysis. Generalized linear mixed effects model with the frequency of offensive behaviors of the puppy-puppy dyads as the response variable. ‘Relation’ of the individual to its dyadic partner (e.g. whether they were ‘subordinate’ or ‘dominant’), ‘sex mix’ of the dyad, and the ‘pack type’ for the dyad (e.g. ‘puppy pack’ versus ‘mixed-age pack’) were predictor variables. An interaction between ‘relation’ and ‘pack type’ was included. Statistics are given for each variable when they were last in the model.
(DOCX)

S4 Table Outputs from the Model 4 analysis. Linear model with the win ratios of the dyads from the mixed-age packs as the response variable with ‘sex mix’ of the dyad, ‘play duration’ for the dyad, as well as ‘age mix’ of the dyad (e.g. ‘puppy-puppy’ versus ‘puppy-adult’) as the predictor variables. Statistics are given for each variable when they were last in the model.
(DOCX)

S5 Table Outputs from the Model 5 analysis. Generalized linear mixed effects model with the frequency of self-handicapping behaviors of the dyads in the mixed-age packs as the response variable. ‘Relation’ of the individual to its dyadic partner (e.g. whether they were ‘subordinate’ or ‘dominant’), ‘sex mix’ of the dyad, as well as ‘age mix’ of the dyad (e.g. ‘puppy-puppy’ versus ‘puppy-adult’) were predictor variables. An interaction between ‘relation’ and ‘age mix’ was included. Statistics are given for each variable when they were last in the model.
(DOCX)

S6 Table Outputs from the Model 6 analysis. Generalized linear mixed effects model with the frequency of offensive behaviors of the dyads in the mixed-age packs as the response variable. ‘Relation’ of the individual to its dyadic partner (e.g. whether they were ‘subordinate’ or ‘dominant’), ‘sex mix’ of the dyad, as well as ‘age mix’ of the dyad (e.g. ‘puppy-puppy’ versus ‘puppy-adult’) were predictor variables. An interaction between ‘relation’ and ‘age mix’ was included. Statistics are given for each variable when they were last in the model.
(DOCX)

S7 Table Dominance & Reversed Submission Behaviors for Puppy Pack 2009. Actors are on the rows while receivers are on the columns.
(DOCX)

S8 Table Dominance & Reversed Submission Behaviors for Kaspar 2009. Actors are on the rows while receivers are on the columns.
(DOCX)

S9 Table Dominance & Reversed Submission Behaviors for Puppy Pack 2012. Actors are on the rows while receivers are on the columns.
(DOCX)

S10 Table Dominance & Reversed Submission Behaviors for Geronimo 2012. Actors are on the rows while receivers are on the columns.
(DOCX)

S11 Table Dominance & Reversed Submission Behaviors for Nanuk 2012. Actors are on the rows while receivers are on the columns.
(DOCX)

S12 Table Dominance & Reversed Submission Behaviors for Kaspar 2012. Actors are on the rows while receivers are on the columns.
(DOCX)
