# Supplementary material for: Play Behavior in Wolves: Using the ‘50:50’ Rule to Test for Egalitarian Play Styles
Source: PLoS One. 2016 May 11;11(5):e0154150. doi: 10.1371/journal.pone.0154150 (PMC4864279; doi:10.1371/journal.pone.0154150)
Supplement: S1 Table — Linear mixed effects model with the win ratios of the puppy-puppy dyads as the response variable with ‘pack type’ (e.g. ‘puppy pack’ versus ‘mixed-age pack’), ‘sex mix’ of the dyad, and the ‘play duration’ for the dyad as the predictor variables. Statistics are given for each variable when they were last in the model. (DOCX) [file pone.0154150.s003.docx]

**S1 Table. Outputs from the Model 1 analysis.** Linear mixed effects model with the win ratios of the puppy-puppy dyads as the response variable with ‘pack type’ (e.g. ‘puppy pack’ versus ‘mixed-age pack’), ‘sex mix’ of the dyad, and the ‘play duration’ for the dyad as the predictor variables. Statistics are given for each variable when they were last in the model.

| **Variable** | **Degrees of Freedom** | **Chisq** | **Proc Logistic** |
| --- | --- | --- | --- |
| Pack Type | 1 | 0.0683 | 0.7937804 |
| Sex Mix | 2 | 0.9203 | 0.6312 |
| Play Duration | 1 | 0.3312 | 0.565 |
