# Supplementary material for: Play Behavior in Wolves: Using the ‘50:50’ Rule to Test for Egalitarian Play Styles
Source: PLoS One. 2016 May 11;11(5):e0154150. doi: 10.1371/journal.pone.0154150 (PMC4864279; doi:10.1371/journal.pone.0154150)
Supplement: S3 Table — Generalized linear mixed effects model with the frequency of offensive behaviors of the puppy-puppy dyads as the response variable. ‘Relation’ of the individual to its dyadic partner (e.g. whether they were ‘subordinate’ or ‘dominant’), ‘sex mix’ of the dyad, and the ‘pack type’ for the dyad (e.g. ‘puppy pack’ versus ‘mixed-age pack’) were predictor variables. An interaction between ‘relation’ and ‘pack type’ was included. Statistics are given for each variable when they were last in the model. (DOCX) [file pone.0154150.s005.docx]

**S3 Table. Outputs from the Model 3 analysis.** Generalized linear mixed effects model with the frequency of offensive behaviors of the puppy-puppy dyads as the response variable. ‘Relation’ of the individual to its dyadic partner (e.g. whether they were ‘subordinate’ or ‘dominant’), ‘sex mix’ of the dyad, and the ‘pack type’ for the dyad (e.g. ‘puppy pack’ versus ‘mixed-age pack’) were predictor variables. An interaction between ‘relation’ and ‘pack type’ was included. Statistics are given for each variable when they were last in the model.

| **Variable** | **Degrees of Freedom** | **Chisq** | **Proc Logistic** |
| --- | --- | --- | --- |
| Relation * Pack Type | 1 | 0.0210 | 0.88478 |
| Relation | 1 | 0.0220 | 0.882157 |
| Sex Mix | 2 | 3.5912 | 0.166031 |
| Pack Type | 1 | 7.6898 | 0.005553 |
