# Supplementary material for: Play Behavior in Wolves: Using the ‘50:50’ Rule to Test for Egalitarian Play Styles
Source: PLoS One. 2016 May 11;11(5):e0154150. doi: 10.1371/journal.pone.0154150 (PMC4864279; doi:10.1371/journal.pone.0154150)
Supplement: S4 Table — Linear model with the win ratios of the dyads from the mixed-age packs as the response variable with ‘sex mix’ of the dyad, ‘play duration’ for the dyad, as well as ‘age mix’ of the dyad (e.g. ‘puppy-puppy’ versus ‘puppy-adult’) as the predictor variables. Statistics are given for each variable when they were last in the model. (DOCX) [file pone.0154150.s006.docx]

**S4 Table. Outputs from the Model 4 analysis.** Linear model with the win ratios of the dyads from the mixed-age packs as the response variable with ‘sex mix’ of the dyad, ‘play duration’ for the dyad, as well as ‘age mix’ of the dyad (e.g. ‘puppy-puppy’ versus ‘puppy-adult’) as the predictor variables. Statistics are given for each variable when they were last in the model.

| **Variable** | **Degrees of Freedom** | **Sum of Squares** | **F Value** | **Proc Logistic** |
| --- | --- | --- | --- | --- |
| Play Duration | 1 | 0.02024 | 0.2906 | 0.594830 |
| Sex Mix | 2 | 0.43743 | 3.2312 | 0.056475 |
| Age Mix | 1 | 0.6468 | 8.2003 | 0.008005 |
