# Supplementary material for: Play Behavior in Wolves: Using the ‘50:50’ Rule to Test for Egalitarian Play Styles
Source: PLoS One. 2016 May 11;11(5):e0154150. doi: 10.1371/journal.pone.0154150 (PMC4864279; doi:10.1371/journal.pone.0154150)
Supplement: S6 Table — Generalized linear mixed effects model with the frequency of offensive behaviors of the dyads in the mixed-age packs as the response variable. ‘Relation’ of the individual to its dyadic partner (e.g. whether they were ‘subordinate’ or ‘dominant’), ‘sex mix’ of the dyad, as well as ‘age mix’ of the dyad (e.g. ‘puppy-puppy’ versus ‘puppy-adult’) were predictor variables. An interaction between ‘relation’ and ‘age mix’ was included. Statistics are given for each variable when they were last in the model. (DOCX) [file pone.0154150.s008.docx]

**S6 Table. Outputs from the Model 6 analysis.** Generalized linear mixed effects model with the frequency of offensive behaviors of the dyads in the mixed-age packs as the response variable. ‘Relation’ of the individual to its dyadic partner (e.g. whether they were ‘subordinate’ or ‘dominant’), ‘sex mix’ of the dyad, as well as ‘age mix’ of the dyad (e.g. ‘puppy-puppy’ versus ‘puppy-adult’) were predictor variables. An interaction between ‘relation’ and ‘age mix’ was included. Statistics are given for each variable when they were last in the model.

| **Model** | **Variable** | **Degrees of Freedom** | **Chisq** | **Proc Logistic** |
| --- | --- | --- | --- | --- |
| Full Model | Sex Mix | 2 | 1.7405 | 0.4188493 |
|  | Relation | 1 | 32.994 | 9.244e-09 |
|  | Age Mix | 1 | 14.173 | 0.0001667 |
|  | Relation * Age Mix | 1 | 28.666 | 8.602e-08 |
| Age Mix : “AP” | Relation | 1 | 31.048 | 2.518e-08 |
| Age Mix : “PP” | Relation | 1 | 1.0251 | 0.3113 |
