# Supplementary material for: Play Behavior in Wolves: Using the ‘50:50’ Rule to Test for Egalitarian Play Styles
Source: PLoS One. 2016 May 11;11(5):e0154150. doi: 10.1371/journal.pone.0154150 (PMC4864279; doi:10.1371/journal.pone.0154150)
Supplement: S7 Table — Actors are on the rows while receivers are on the columns. (DOCX) [file pone.0154150.s009.docx]

**S7 Table. Dominance & Reversed Submission Behaviors for Puppy Pack 2009.** Actors are on the rows while receivers are on the columns

|  | **Nanuk** | **Geronimo** | **Apache** | **Yukon** | **Tatonga** | **Cherokee** |
| --- | --- | --- | --- | --- | --- | --- |
| **Nanuk** | 0 | 38 | 7 | 10 | 16 | 17 |
| **Geronimo** | 0 | 0 | 2 | 2 | 7 | 4 |
| **Apache** | 0 | 2 | 0 | 7 | 4 | 4 |
| **Yukon** | 0 | 0 | 4 | 0 | 10 | 2 |
| **Tatonga** | 0 | 1 | 3 | 5 | 0 | 13 |
| **Cherokee** | 0 | 0 | 6 | 0 | 10 | 0 |
