# Supplementary material for: Play Behavior in Wolves: Using the ‘50:50’ Rule to Test for Egalitarian Play Styles
Source: PLoS One. 2016 May 11;11(5):e0154150. doi: 10.1371/journal.pone.0154150 (PMC4864279; doi:10.1371/journal.pone.0154150)
Supplement: S8 Table — Actors are on the rows while receivers are on the columns. (DOCX) [file pone.0154150.s010.docx]

**S8 Table. Dominance & Reversed Submission Behaviors for Kaspar 2009.** Actors are on the rows while receivers are on the columns.

|  | **Kaspar** | **Aragorn** | **Shima** | **Nanuk** | **Geronimo** | **Yukon** | **Cherokee** | **Apache** | **Tatonga** |
| --- | --- | --- | --- | --- | --- | --- | --- | --- | --- |
| **Kaspar** | 0 | 13 | 6 | 3 | 14 | 42 | 42 | 49 | 26 |
| **Aragorn** | 1 | 0 | 17 | 10 | 25 | 53 | 26 | 32 | 92 |
| **Shima** | 0 | 0 | 0 | 8 | 10 | 23 | 6 | 40 | 39 |
| **Nanuk** | 4 | 0 | 0 | 0 | 25 | 3 | 16 | 19 | 3 |
| **Geronimo** | 5 | 1 | 2 | 7 | 0 | 5 | 9 | 8 | 9 |
| **Yukon** | 0 | 2 | 1 | 0 | 0 | 0 | 4 | 5 | 2 |
| **Cherokee** | 0 | 1 | 1 | 0 | 1 | 0 | 0 | 3 | 2 |
| **Apachee** | 0 | 0 | 0 | 1 | 1 | 1 | 2 | 0 | 3 |
| **Tatonga** | 2 | 0 | 1 | 0 | 0 | 0 | 0 | 0 | 0 |
