# Supplementary material for: Play Behavior in Wolves: Using the ‘50:50’ Rule to Test for Egalitarian Play Styles
Source: PLoS One. 2016 May 11;11(5):e0154150. doi: 10.1371/journal.pone.0154150 (PMC4864279; doi:10.1371/journal.pone.0154150)
Supplement: S9 Table — Actors are on the rows while receivers are on the columns. (DOCX) [file pone.0154150.s011.docx]

**S9 Table. Dominance & Reversed Submission Behaviors for Puppy Pack 2012.** Actors are on the rows while receivers are on the columns.

|  | **Tala** | **Chitto** | **Una** | **Amarok** | **Wamblee** | **Kay** |
| --- | --- | --- | --- | --- | --- | --- |
| **Tala** | 0 | 3 | 7 | 2 | 5 | 8 |
| **Chitto** | 0 | 0 | 2 | 13 | 2 | 3 |
| **Una** | 7 | 0 | 0 | 2 | 3 | 3 |
| **Amarok** | 6 | 2 | 1 | 0 | 4 | 2 |
| **Wamblee** | 0 | 0 | 0 | 0 | 0 | 5 |
| **Kay** | 0 | 0 | 0 | 0 | 1 | 0 |
