# Supplementary material for: Play Behavior in Wolves: Using the ‘50:50’ Rule to Test for Egalitarian Play Styles
Source: PLoS One. 2016 May 11;11(5):e0154150. doi: 10.1371/journal.pone.0154150 (PMC4864279; doi:10.1371/journal.pone.0154150)
Supplement: S11 Table — Actors are on the rows while receivers are on the columns. (DOCX) [file pone.0154150.s013.docx]

**S11 Table. Dominance & Reversed Submission Behaviors for Nanuk 2012.** Actors are on the rows while receivers are on the columns.

|  | **Nanuk** | **Yukon** | **Una** | **Wamblee** |
| --- | --- | --- | --- | --- |
| **Nanuk** | 0 | 0 | 10 | 5 |
| **Yukon** | 0 | 0 | 2 | 7 |
| **Una** | 0 | 0 | 0 | 3 |
| **Wamblee** | 0 | 0 | 1 | 0 |
