# Supplementary material for: Play Behavior in Wolves: Using the ‘50:50’ Rule to Test for Egalitarian Play Styles
Source: PLoS One. 2016 May 11;11(5):e0154150. doi: 10.1371/journal.pone.0154150 (PMC4864279; doi:10.1371/journal.pone.0154150)
Supplement: S12 Table — Actors are on the rows while receivers are on the columns. (DOCX) [file pone.0154150.s014.docx]

**S12 Table. Dominance & Reversed Submission Behaviors for Kaspar 2012.** Actors are on the rows while receivers are on the columns.

|  | **Kaspar** | **Aragorn** | **Shima** | **Tala** | **Chitto** |
| --- | --- | --- | --- | --- | --- |
| **Kaspar** | 0 | 2 | 2 | 8 | 19 |
| **Aragorn** | 0 | 0 | 1 | 4 | 7 |
| **Shima** | 0 | 0 | 0 | 21 | 3 |
| **Tala** | 0 | 0 | 0 | 0 | 1 |
| **Chitto** | 0 | 0 | 0 | 1 | 0 |
